# Prospective Genetic Screening in Multiple Endocrine Neoplasia Syndromes

**DOI:** 10.3390/children11081012

**Published:** 2024-08-20

**Authors:** Diana Paun, Dana Tilici, Sorin Paun, Alexandra Mirica

**Affiliations:** 1 The Faculty of Medicine, Carol Davila University of Medicine and Pharmacy, 020021 Bucharest, Romania; diana-loreta.paun@umfcd.ro (D.P.); sorin.paun@umfcd.ro (S.P.); alexandra.mirica@umfcd.ro (A.M.); 2 Endocrinology Department, Emergency University Hospital Bucharest, 050098 Bucharest, Romania; 3 Clinical Emergency Hospital Bucharest, 014461 Bucharest, Romania; 4 Grigore Alexandrescu Emergency Hospital for Children, 010621 Bucharest, Romania

**Keywords:** multiple endocrine neoplasia syndromes, screening, early diagnosis, genetic screening

## Abstract

Multiple endocrine neoplasia syndromes are a rare but potentially fatal pathology due to the lack of early diagnosis. We have performed a narrative review of the medical literature, summarizing the main clinical concepts useful in current clinical practice, showing the importance of screening and early diagnosis during childhood.

## 1. Multiple Endocrine Neoplasia Type 1 (MEN1) Syndrome

### 1.1. Clinical Overview

Multiple endocrine neoplasia type 1 (*MEN1*) stands as a rare, hereditary syndrome of endocrine tumors characterized by an autosomal dominant pattern of inheritance, deriving from mutations within the *MEN1* gene. Situated on the lengthy arm of chromosome 11 (11q13), this gene serves as a tumor suppressor, overseeing the production of the *menin* protein. *Menin*, within its functional domain, assumes multifaceted responsibilities in coordinating cellular division, proliferation, transcriptional regulation, and maintaining genomic stability [1,2].

The *MEN1* gene exhibits considerable penetrance, enabling its diagnosis through a spectrum of clinical, familial, or genetic presentations. The syndrome is marked by an autosomal dominant mode of inheritance, featuring an exceptionally heightened penetrance—50% of affected individuals present with distinctive tumors by the age of 20, rising to 95% by the age of 40 [2].

The global prevalence of MEN1 syndrome is estimated to be 1 in 30,000 individuals. Interestingly, no clear predilection has been observed concerning sex, age, or gender among MEN1 patients. Diagnosis of MEN1 typically requires the presence of a minimum of two out of the three classic tumor subtypes originating from the pituitary, pancreatic, and parathyroid glands. Additionally, MEN1 syndrome is associated with a diverse spectrum of approximately 20 endocrine and non-endocrine tumors, highlighting the complexity and variability of its clinical manifestations [3].

In children, the primary expression of MEN1 syndrome often presents as primary hyperparathyroidism (PHPT) due to parathyroid gland hyperplasia, affecting up to 75% of cases, typically emerging after the age of 10. By age 50, approximately 95% of pediatric MEN1 patients develop PHPT, with onset usually between 20 and 25 years of age, a notably early occurrence compared to sporadic forms [1].

MEN1-related PHPT is distinguished by diffuse parathyroid hyperplasia, contrasting with the single adenomas commonly observed in sporadic PHPT. Despite the presence of hypercalcemia, symptoms are frequently absent, though affected children might exhibit malaise, polyuria, polydipsia, or constipation. Instances of severe hypercalcemia leading to crises are rare in this pediatric context [1,4].

Duodenopancreatic neuroendocrine tumors (NETs) are the second most common manifestation of MEN1 syndrome. Typically small (<2 cm) and often multiple, these tumors can present with hormonal syndromes or remain non-functioning. Approximately 20% of MEN1 patients develop NETs as their first symptom, which can appear as early as age 5 (e.g., insulinoma). These tumors are the leading cause of death due to malignancy. The presence of multiple hormonal secretion in pancreatic NETs among MEN1 patients is associated with improved survival, possibly due to routine screening. While small pancreatic NETs (<2 cm) usually remain stable over the long term, some may progress slowly [5].

Gastrinomas, non-functioning pancreatic polypeptide-secreting tumors (PPomas), insulinomas, glucagonomas, and vasoactive intestinal polypeptidomas (VIPomas) constitute the spectrum of NETs associated with MEN1, with varying degrees of penetrance [5].

Gastrinomas, which cause Zollinger–Ellison syndrome (ZES), are the most common functional duodenopancreatic neuroendocrine tumors (NETs) in MEN1, affecting 21–70% of patients. They rarely appear as the first symptom of MEN1 (8% of cases). The youngest reported case of ZES in MEN1 was a 7-year-old girl. Most MEN1-related gastrinomas (>80%) are found in the duodenum, often with non-functioning pancreatic NETs, complicating diagnosis and treatment. This underlines the importance of genetic screening for MEN1 in individuals presenting with ZES [5,6].

Insulinomas are detected in roughly 20% of MEN1 patients, serving as the initial clinical sign in about 10% of cases. These tumors tend to emerge early in MEN1, even earlier than gastrinomas, with the youngest documented case found in a 5-year-old girl. In individuals with MEN1, roughly 8% of insulinomas are malignant [5].

Additionally, pancreatic NETs are uncommon in MEN1, affecting less than 2% of patients. Typically, they present as large tumors (>3 cm) already metastasized to nearby lymph nodes or the liver. 

In MEN1 syndrome, glucagonomas are rare, found in less than 2% of cases, and present similarly to sporadic cases, including weight loss and diabetes mellitus.

VIPomas, responsible for Verner–Morrison syndrome, are very rare in MEN1. Few cases of duodenal or pancreatic somatostatin-producing NETs have been reported in MEN1, mostly without somatostatinoma syndrome.

Additionally, pancreatic NETs may secrete growth-hormone-releasing hormone (leading to acromegaly), parathyroid-hormone-related protein, or adrenocorticotropic hormone (ACTH) [5].

Non-functioning pancreatic neuroendocrine tumors (NETs) have emerged as the predominant subtype among pancreatic NETs in MEN1 syndrome. Recent systematic endoscopic ultrasonography (EUS) studies in MEN1 individuals devoid of pancreatic symptoms have unveiled that 55% harbor non-functioning pancreatic NETs. Intriguingly, these neoplasms have been detected in asymptomatic adolescents as young as 12 years old. The cohort of non-functioning pancreatic NETs includes authentic non-secreting tumors, as well as those with hormone secretion insufficient to provoke a clinical syndrome (e.g., glucagonomas) or they are not associated with a recognizable hormonal syndrome (e.g., pancreatic polypeptide) [5].

Pituitary adenomas rank as the third most common tumor in MEN1 adults. They are present in 15–50% of individuals with MEN1 syndrome, seldom appearing as the initial indication of the syndrome, particularly in the sporadic form (around 15% of cases) and less than 10% in the familial form. Pituitary tumors associated with MEN1 mutations tend to be larger, more aggressive, and resistant to conventional therapies. They predominantly manifest as macroadenomas (85%), compared to sporadic cases (42% above 1 cm).

Clinical presentations are characteristic of specific hormone overproduction. Headaches or visual field defects are more common in MEN1-related tumors. These tumors occur more frequently in women and at younger ages compared to sporadic cases. MEN1-related tumors may more often secrete multiple hormones, leading to challenges in diagnosis and treatment. Mixed tumors, particularly prolactin- and adrenocorticotropin-secreting tumors, are more prevalent in MEN1-related cases. In addition to this, multiple tumors may be observed, resembling pituitary hyperplasia seen in autopsy studies of patients with MEN1 mutations. Histopathological features do not reliably distinguish between sporadic and MEN1-related pituitary tumors. Hyperplasia may result from ectopic production of liberins, leading to clinical and biochemical indicators of conditions such as acromegaly, even in the absence of pituitary lesions. Despite their tendency to be more invasive and less responsive to treatment, MEN1-related pituitary tumors do not show a higher incidence of malignant transformation compared to sporadic tumors [7].

In children, pituitary adenomas are the second most frequent MEN1 tumor, with an onset as early as 5 years of age. Somatotrophinomas and corticotrophinomas, along with non-functioning adenomas, constitute other anterior pituitary tumors observed in MEN1 patients, with clinical manifestations dependent on hormone secretion and tumor size [1].

Infrequently, neuroendocrine neoplasms (NENs) connected to MEN1 are diagnosed in diverse anatomical sites, including the stomach, thymus, lung, bronchopulmonary tract (in 3–10% of cases), and, more recently, in the female breast (about 7%) [1,2,5].

Beyond these, MEN1 individuals may develop various non-endocrine lesions, such as lipomas (30%), collagenomas (70%), and facial angiofibromas (85%) [8,9].

### 1.2. Diagnosis

The diagnosis of MEN1 syndrome hinges upon a thorough consideration of clinical manifestations, familial history, and genetic markers.

Firstly, diagnosis of MEN1 syndrome can be made if a patient has at least two primary endocrine tumors associated with MEN1. These can include parathyroid adenomas, enteropancreatic tumors, and pituitary adenomas.

Secondly, the development of a MEN1-associated tumor in a first-degree relative of an individual already diagnosed with MEN1 serves as another path to diagnosis. This scenario shows the hereditary nature of MEN1, emphasizing the importance of familial screening and genetic counseling. Lastly, the identification of a germline MEN1 mutation in an asymptomatic individual, without biochemical or radiological evidence of MEN1, can lead to a definitive diagnosis. This genetic marker, even in the absence of overt clinical signs, points toward the potential future development of MEN1-related endocrine neoplasms. For relatives of individuals with MEN1, it is essential to undergo genetic testing before the age of 5 due to the asymptomatic nature of the condition during childhood. Around 5–25% of those clinically diagnosed with MEN1 lack identifiable mutations, complicating testing. Confirmed *MEN1* mutation carriers often have a reduced lifespan, averaging 63 years, primarily due to gastroenteropancreatic malignancies [1].

Recent studies highlight the importance of genetic screening for familial MEN1, as it can lead to diagnoses up to a decade earlier than traditional clinical or biochemical methods, providing a critical opportunity for proactive management and monitoring of this complex endocrine disorder [10].

### 1.3. Genetics

The *MEN1* gene, located on chromosome 11q13, spans 9 kilobases and includes 10 exons that code for the 610-amino acid menin protein. An alternative splice site in exon 2 produces a 615-amino acid variant. *Menin* is widely expressed and primarily found in the nucleus of resting cells. The *MEN1* gene contains three nuclear localization signals in its C-terminal region and two nuclear export signals in the N-terminal and middle regions, regulating *menin*’s movement between the nucleus and cytoplasm [5].

*MEN1* acts as a tumor suppressor gene and follows an autosomal dominant inheritance pattern. However, inheriting one defective *MEN1* allele alone is not enough to cause tumorigenesis—an additional event affecting the remaining normal allele is required. Tumor DNA analysis in about 90% of cases shows loss of heterozygosity of the normal *MEN1* allele compared to leukocyte DNA. This loss is consistently observed across various MEN1-related tumors, including those of the parathyroid, pancreatic, pituitary, adrenal, thymic, and bronchopulmonary systems. Research highlights *menin*’s diverse functions in cellular processes, including transcriptional regulation, chromatin organization, growth factor and hormone signaling, and cytoskeletal dynamics [11].

Mutation analyses have shown frequent mutations (>75% of cases) in MEN1 among afflicted individuals. Notably, over 10% of *MEN1* mutations arise de novo, potentially transmissible to subsequent generations. In cases where standard sequencing fails to discern mutations within the coding region, adjunctive methodologies are warranted to detect large deletions (affecting 2.5–4% of patients), intronic aberrations, genetic rearrangements, or anomalies within the non-coding exon 1, typically addressed via multiplex ligation-dependent probe amplification.

Currently, the *MEN1* population exhibits a heterogeneous array of over 1300 unique germline or somatic mutations, coupled with the identification of 24 MEN1 polymorphisms. A definitive genotype–phenotype correlation remains elusive, likely due to the essential role of menin’s various protein domains in maintaining its functional integrity [5]. Research has explored potential epigenetic modifications in *menin*, which may contribute to *MEN1* phenocopies, especially in mutation-negative cases. MicroRNAs (miRNAs) are key regulators of *menin* expression, with some associated with the development of insulinomas and parathyroid adenomas. Additionally, hypermethylation of CpG regions is common in both MEN1-related and sporadic neuroendocrine tumors (NETs). The reversible nature of epigenetic changes suggests potential for developing new therapeutic approaches [1,12].

### 1.4. Screening Approaches

The identification of the causative gene for MEN1 syndrome has revolutionized its clinical management, profoundly impacting the reduction in disease-associated morbidity and mortality. Adherence to recommended guidelines for genetic counseling and germline *MEN1* genetic testing has significantly enhanced the precision of disease prognosis in index cases and their familial cohorts [13].

In MEN1 patients, mortality rates linked to syndrome manifestations can reach up to 50%, with pancreatic endocrine tumors being the predominant cause of death. Hence, timely screening is imperative to alleviate potential adverse consequences [13,14].

Around 12% to 17% of MEN1 patients are identified with the condition within the first two decades of life [8].

Current screening recommendations are guided by the earliest reported ages of respective manifestations and incorporate considerations for rare adverse outcomes in pediatric populations. Thus, clinical and biochemical screening is advocated annually in at-risk children for MEN1 from approximately 5 years of age, with regular radiological imaging of the pancreas and pituitary beginning around age 10. However, uncertainties persist regarding the value of screening for detecting presymptomatic disease, particularly as most clinically significant early-onset tumors manifest symptomatically. Nevertheless, in cases such as prolactinoma, insulinoma, or Cushing disease where symptoms arise, investigation should not be delayed. The role of early intervention for asymptomatic tumors, such as biochemical primary hyperparathyroidism, remains ambiguous, despite its penetrance reaching 50–75% by age 21. Similarly, the optimal age to commence pancreatic imaging for non-functioning pancreatic NETs (NF-PNETs) is debatable, with recent studies suggesting varying risks of clinically relevant tumors at different ages. Balancing potential screening burdens and risks with the likelihood of “missing” a clinically significant tumor is pivotal in determining the screening initiation age. For asymptomatic young children, deferring investigation until the mid-teenage years may be considered prudent, with a less frequent screening regimen possibly suitable thereafter [13,15].

Regarding NF-PNETs, surgery is typically considered for tumors larger than 1 cm, although evidence supporting this recommendation is scant. Notably, the risk of metastatic disease significantly escalates for tumors exceeding 2 cm, suggesting size-based management decisions, with a 2 cm threshold often endorsed for surgical intervention. Therefore, screening’s primary objective should be the detection of clinically significant tumors, while the utility of detecting smaller NF-PNETs (<1 cm) remains uncertain due to the unlikelihood of intervention. Current guidelines recommend annual imaging modalities, such as CT, MRI, or EUS, for tumors > 1 cm, prioritizing safety and acceptability, with periodic MRI seemingly posing the lowest harm potential, especially when utilizing non-contrast diffusion-weighted imaging sequences. Considering the slow evolution of most NF-PNETs, less frequent surveillance intervals (e.g., every 2–3 years) may suffice for patients with consistently negative imaging, although a small subset may still develop metastatic disease, necessitating tailored risk stratification and potential adaptation of surveillance strategies [15].

For bronchopulmonary (BP) and thymic NETs, current MEN1 guidelines advise imaging every 1–2 years with CT or MRI, albeit acknowledging the limited evidence supporting this approach. Recent studies reveal radiological evidence of BP NETs in approximately 25% of MEN1 patients, often exhibiting slow growth rates and favorable overall survival, casting doubt on the necessity of frequent screening in asymptomatic individuals, particularly for small, stable lesions. Similarly, thymic NETs, although rare, are associated with aggressive disease courses and high mortality rates, warranting frequent CT or MRI scans every 1–2 years for early detection, although the number of scans needed for asymptomatic patients remains high. Decisions regarding screening frequency should thus be individualized, with MRI preferred over CT to mitigate cumulative radiation exposure risks [15].

It is suggested that individuals with a *MEN1* germline mutation undergo annual screenings for tumors related to *MEN1* (as outlined in Table 1).

### 1.5. Management Strategies

The primary therapeutic strategy for parathyroid tumors in MEN1 syndrome is surgical resection, although the optimal timing and surgical techniques remain subjects of ongoing debate, particularly in pediatric and adolescent populations. Surgical options include partial parathyroidectomy, subtotal parathyroidectomy (removal of 3.5 glands), or total parathyroidectomy with or without auto-transplantation. Postoperative complications, such as permanent hypoparathyroidism and laryngeal nerve damage, necessitate careful consideration based on the patient’s age and timing of the surgery.

Although research on MEN1-related primary hyperparathyroidism (PHPT) is limited, evidence indicates favorable outcomes with partial parathyroidectomy in children, aligning with findings in adults with non-MEN1 PHPT. Persistent PHPT can impair bone health, raising the risk of osteoporosis and fractures, underscoring the need for surgical intervention to prevent long-term complications. Recent studies emphasize refining surgical techniques and postoperative care to improve outcomes for MEN1 patients with PHPT [9,16].

Pancreatic neuroendocrine neoplasms (pNENs) present a management challenge due to their variability, from indolent to aggressive forms. Advances in imaging have improved detection, with up to 60% of cases identified when tumors are <2 cm. Many of these small tumors remain stable over a decade, with a 5-year survival rate near 100% for well-differentiated, non-functioning pNENs < 2 cm. For tumors < 1 cm, active surveillance is often sufficient, while those of 1–2 cm require consideration of patient age, comorbidities, tumor location, and growth. Larger tumors or those with hormonal hypersecretion generally need prompt surgical intervention to remove the tumor while preserving pancreatic function. Lymph node metastasis affects surgical planning and outcomes.

Localization techniques, including ultrasonography, are crucial for identifying tumors, especially in non-localized functioning pNENs. Liver metastases are challenging; while surgical cytoreduction can help, complete resection is often impractical. A multidisciplinary approach is used to manage these cases, combining surgical and radiological techniques to control the tumor and alleviate symptoms. For patients with multiple endocrine neoplasia type 1 (MEN1) syndrome, the management of pNENs is complicated by the presence of multifocal tumors and other endocrines, and a more comprehensive approach is necessary to address the disease’s multifaceted nature.

Beyond surgical interventions, interventional therapies, such as ethanol injection and radiofrequency ablation, are promising for small tumors. Systemic therapies, including somatostatin analogues and tyrosine kinase inhibitors, offer additional options, particularly for advanced or metastatic pNENs. However, the efficacy and safety of these treatments in the context of MEN1-associated pNENs require further investigation through prospective studies focusing on this patient population [6].

The primary therapeutic goal for patients with symptomatic functioning pancreatic neuroendocrine tumors (NETs), including insulinomas, is surgical resection with curative intent. 

Management of gastrinomas presents challenges. Non-metastasizing pancreatic gastrinomas may be amenable to curative surgery, ideally performed by an experienced endocrine surgeon. In MEN1 patients, where multiple small duodenal gastrinomas are common, treatment becomes more complicated. For most, proton-pump inhibitors are the standard medical approach. However, specialized centers might consider options such as local excision, duodenectomy, or occasionally duodenopancreatectomy, depending on patient preferences, as these methods could improve cure rates. 

Medical management typically includes proton-pump inhibitors and somatostatin analogs to control hyperacidity. Regular gastroscopic surveillance is recommended for monitoring hypergastrinemia and detecting potential peptic ulcer disease or gastric carcinoid tumors.

The surgical management of non-functioning pancreatic tumors remains debated. Surgical resection is advised for tumors exceeding 1 cm in size or those showing significant growth within 6–12 months.

For non-resectable tumor masses, therapeutic options include somatostatin analogs, biotherapy, targeted radionuclide therapy, locoregional treatments, and chemotherapy. Chemotherapy is utilized for inoperable or metastatic pancreatic NETs, with agents such as sunitinib and everolimus considered for advanced, progressive, well-differentiated pancreatic NETs.

In the context of pituitary tumors, annual biochemical screening, including plasma prolactin and IGF-I levels, and pituitary MRI every 3–5 years, are advised based on clinical judgment and available resources. Abnormal findings necessitate further hypothalamic–pituitary testing to elucidate the nature of the pituitary lesion and its impact on other pituitary hormone secretions [16].

The management of pituitary tumors in patients with a confirmed *MEN1* gene mutation follows standard treatment protocols. This typically includes the use of cabergoline or bromocriptine for prolactinomas, and neurosurgical resection for somatotropinomas. Depending on the patient’s clinical condition, additional radiotherapy may be required for residual tumor masses.

Patients with MEN1-related pituitary tumors tend to have a less favorable response to these standard treatments, with normalization of pituitary hormone levels observed in only 42% of MEN1 cases, compared to a 90% success rate in patients with sporadic pituitary tumors [7].

## 2. Multiple Endocrine Neoplasia Type 2 (MEN2)

Multiple endocrine neoplasia type 2 (MEN2) is an autosomal dominant condition, further divided into 2A (MEN2A) and 2B (MEN2B), formerly known as MEN3, which are two separate disorders, in terms of tumors that might develop, treatment principles, and long-term monitoring. Furthermore, there are four variations of MEN2A: classical MEN2A, MEN2A with Hirschsprung disease (HD), MEN2A with cutaneous lichen amyloidosis (CLA), and familial medullary thyroid carcinoma (FMTC) [18].

MEN2B is characterized by pheochromocytoma (PHEO) and medullary thyroid carcinoma (MTC), but not hyperparathyroidism (HPT), associating other clinical features, such as marfanoid habitus, mucosal neuromas, and digestive implications [19].

In terms of epidemiology, there are 1 in 35,000 cases of all MEN2 globally, compared to 1 in 30,000–50,000 cases in the US. It is described that 1 in 600,000 patients have MEN2B syndrome. In addition, MEN2A syndrome affects 60% to 90% of MEN2 families, while MEN2B syndrome has been described in about 5% of MEN2 families [1].

Regarding the etiology of multiple endocrine neoplasia type 2 (MEN2) syndrome, the responsible genetic mutation is represented by the activating mutation of the receptor tyrosine kinase, also known as the rearranged during transfection (*RET)* protein, which is located on chromosome 10q. This protein transduces molecular signals related to cell development and differentiation in tissues of embryonic ectodermal origin. Mutations can affect both extracellular and intracellular domains of the *RET* signaling pathway [20].

It is worth mentioning that this genetic modification is activating, compared to most mutations responsible for the development of neoplasia, which are mostly inactivating or associated with a “loss of function”, thus leading to inactivation of tumor suppressor proteins [21].

Studies on familial forms have started to identify several molecular mechanisms that promote tumor formation, such as tyrosine kinase activation, stimulation of hypoxia-inducible factors, activation of *Ras* oncogenes, or impaired apoptosis, even if the precise pathophysiology of PHEO formation is still mostly unclear. These studies have also shown that distinct genetic mutations may have different effects on the secretory biological traits of PHEO. For instance, tumors in VHL are primarily noradrenergic (secreting noradrenaline), whereas MEN2-associated Feo are primarily adrenergic (secreting adrenaline). Conversely, cancers linked to mutations involving SDHx often exhibit a noradrenergic secretory pattern [22].

In addition, looking further into the mechanism, the extracellular portion of this tyrosine kinase *RET* has a ligand-binding domain, a region that resembles cadherin, and a cysteine-rich domain near the cell membrane. Additionally, it also contains an intracellular portion with two tyrosine kinase subdomains, TK1 and TK2, and a single transmembrane domain. MEN2A syndrome can be caused by several distinct mutations. The cysteine-rich region of the extracellular domain of the *RET* protein, which is encoded by the exon 10 and exon 11 genes, is where most mutations causing MEN2A syndrome occur [23].

On the other hand, MEN2B-associated tumors are caused by mutations in the intracellular TK2 domain. For example, mutations in exon 16, involving a single mutation from 918 Met to Thr (M918T), account for more than 95% of the genetic causes of MEN2B syndrome. 

However, less common genetic mutations correlated with MEN2A and MEN2B syndromes are also described, which are classified according to the latest publications as high, moderate, or low risk [18].

Early genetic diagnosis of these particular mutations is crucial, as they greatly impact diagnosis, treatment, prognosis, and tumor screening. 

Moreover, the inheritance pattern for MEN2A and 2B is autosomal dominant and has a very high penetrance. Tumors affecting the thyroid, parathyroid, and adrenal glands have a risk of shortening life expectancy and lowering quality of life. Early detection is crucial for both sporadic and hereditary medullary thyroid cancers (MTCs), as evidenced by the favorable prognosis for MTCs detected at an early stage and a young age [24,25]. In order to comprehend the significant heterogeneity within and between families, a rigorous investigation for genotype–phenotype relationships has been underway since the genetic variations were discovered. This research has effectively enhanced the prognosis risk assessment based on genotype, which has improved patient care through in-depth genetic counseling.

### 2.1. MEN2A

#### 2.1.1. Clinical Picture

Sipple syndrome, also known as multiple endocrine neoplasia type 2 (MEN2), comprises a constellation of unusual familial cancer syndromes affecting many endocrine organs, most commonly the parathyroid, thyroid, and adrenal glands. Sipple originally identified MEN2 in 1961 after observing a strong correlation between bilateral PHEOs and MTC. Subsequently, involvement in various tissues and organs, including the skin and stomach, has been reported. MEN2 has high genetic penetrance and variable phenotypic expressivity. Even though MEN2 is a rare and unusual diagnosis, it is crucial to recognize the pathology early for the evaluation and care of patients as well as affected family members. In terms of pediatrics, the diagnosis of MEN2A is suspected when a child has two or more of the following endocrine manifestations: MTC, PHEO, and/or PHPT, or family members with these pathologies [25].

With 100% penetrance, MTC is the most frequent MEN2A and MEN2B clinical manifestation and typically the initial medical condition in MEN2 patients, frequently diagnosed at a young age. The hyperplasia of calcitonin-producing parafollicular C-cells results in MTC. The most typical presentations of MTC are cervical lymphadenopathy and a single thyroid nodule. Up to 25% of all MTCs are familial, whereas 75% of cases are sporadic. The multicentric and dense MTCs in the upper part of the thyroid gland in MEN2 patients are similar to the usual distribution of parafollicular cells [26].

The highest incidence of index cases in MEN2A-associated MTC occurs in the third decade of life; conversely, in MEN2B, it often occurs earlier. For many mutations, the age at which MTC first appears is becoming younger over time. For instance, mutations in codons 634 and 918 have been reported to manifest at the earliest ages of onset, which are 10 months and 2 months, respectively [18].

When discovered in childhood, MTC is often linked with a good prognosis since it often emerges from pre-existing C-cell hyperplasia. Ninety to ninety-five percent of pediatric MTC cases are MEN2A instances, mostly caused by mutations in RET’s codon 634. The most severe clinical manifestation of MTC is linked to MEN2B, which is nearly invariably caused by the RET mutation Met918Thr [27].

The clinical presentation and features of MEN2-associated MTC are comparable to those of sporadic MTC, assuming the condition is identified as an index case. However, sporadic MTC usually manifests later in life. The most typical clinical manifestation is cervical lymphadenopathy or a single thyroid nodule. Nuclei positioned eccentrically in fine-needle aspiration (FNA) biopsies are bigger and more pleomorphic than those seen in typical follicular cells. The cytoplasm is often arranged as a tear drop or cytoplasmic tail and may occasionally be somewhat grainy [28]. Immunocytologic staining for calcitonin should be carried out if the diagnosis is suspected.

Less frequently occurring presentations unique to MEN2-associated MTC include flushing brought on by the tumor’s secretion of other peptides, or diarrhea brought on by gastrointestinal secretion of fluid and electrolytes, and incidental identification during a clinical screening conducted after an associated disease (such as PHEO or PHPT) becomes apparent. MTC can occasionally result in Cushing syndrome because of ectopic corticotropin (ACTH) production, but reported cases are rare [29,30].

When MTC is detected in asymptomatic people by calcitonin measuring or DNA testing of RET protooncogene, and the diagnosis is made because of a family history of MEN2 or the existence of an accompanying illness, the condition is frequently characterized as C-cell hyperplasia, a preneoplastic state [22]. When a patient has a palpable tumor, their basal serum calcitonin values are nearly always high and typically correspond with tumor mass. The levels may be normal in individuals with small tumors and those with C-cell hyperplasia, but they may rise abnormally upon infusion of calcium or pentagastrin [31].

Moreover, elevation of calcitonin can also be found in chronic renal insufficiency, follicular thyroid tumors, and lymphocytic thyroiditis [32].

Furthermore, forty to fifty percent of individuals with MEN2A or MEN2B develop PHEO, which is frequently bilateral and multicentric. The incidence and penetration of this tumor greatly depend on the particular kind of mutation. It is often discovered during the screening procedure for people who have MEN2 that is known or suspected. While PHEO seldom manifests prior to MTC, it might be the first sign of MEN2 and cause paroxysmal episodes of headache, anxiety, hypertension, diaphoresis, and palpitations, among other clinical characteristic symptoms [33]. PHEO is predominantly diagnosed in individuals aged 25 to 32 years; however, its occurrence has also been documented in younger patients, as early as 8 to 12 years of age [34].

In addition, PHPT is present in 10% to 25% of patients with MEN2A but is not part of the clinical constellation of MEN2B syndrome. The pathology is intricate with mild evolution, with the peak age of diagnosis occurring after the third decade [20]. When hypercalcemia coexists with elevated (or improperly normal) blood parathyroid hormone (PTH) concentrations, the diagnosis of PHP is made.

There is a phenotype–genotype correlation, as primary hyperparathyroidism (PHPT) is more frequently diagnosed in association with the mutation at codon 634 in exon 11 of the *RET* gene. While multiglandular disease in the parathyroids is possible, it is often observed that hyperplasia and adenoma coexist. In some cases, only a single adenoma is identified during the investigation and subsequent surgical intervention. Serum calcium levels should be used to test for PHPT in patients with MEN2 syndromes [35].

An uncommon skin disorder, called cutaneous lichen amyloidosis (CLA), also known as lichen planus amyloidosis, is described in MEN2A. The condition is believed to be a primary neuropathy, characterized by pruritic, pigmented, scaly papules that often appear on the extensor surfaces of the limbs and in the interscapular area. Histology examination of the lesions has revealed the presence of amyloid [31].

MEN2A is also linked to Hirschsprung disease (HD), commonly referred to as chronic aganglionic megacolon. It is distinguished by the lack of autonomic ganglion cells in the sigmoid colon’s parasympathetic chain, which causes megacolon and diminished peristalsis [26].

In the clinical onset of MEN2B syndrome, musculoskeletal implications have also been described. MEN2B is the only disorder linked to marfanoid habitus, kyphoscoliosis or lordosis, joint laxity, mucosal neuromas, usually at the lips and tongue, and intestinal ganglioneuromas [36].

Familial MTC is considered a subtype of MEN2A, representing 25% of all MTC cases [37]. It is defined by the absence of PHEO or PHPT in individuals with MTC or in families where a RET germline mutation is present [28]. It progresses in a very unusual way, with hard-to-identify micro-metastases that frequently stay stable for years before abruptly accelerating uncontrollably. Furthermore, MTC is among the neoplasms with the finest genetic characterization, whether in its familial or sporadic form.

#### 2.1.2. Clinical Presentation in MEN2A

Any patient with a diagnosis of MTC or PHEO should be suspected of having MEN2A and MEN2B, especially if the patient presents at a relatively young age (usually under 35).

The most typical presentations of MTC are cervical lymphadenopathy or a single thyroid nodule. It is recommended that *RET* protooncogene mutations for both MEN2A and MEN2B be checked for in patients with a diagnosis of MTC or a family history of MTC. Tests for MEN2A and MEN2B should be performed on individuals diagnosed with PHEO at an earlier age than those with its sporadic variants [1].

PHEO is commonly characterized by headache paroxysms, anxiety, diaphoresis and palpitations, and elevated blood pressure. Screening for MEN2 should be initiated if these symptoms are present in the third decade, especially between the ages of 25 and 32. It is important to obtain a thorough medical history of any linked conditions (as mentioned above) that the patient or any family members may have. Marfanoid habitus (a reduced upper to lower body ratio), mucosal neuromas (red papules) across the lips and tongue, and joint hyperlaxity linked to MEN2B are other probable physical examination findings. Unlike Marfan syndrome, the individuals with MEN2B usually do not have aortic anomalies or lens dislocation. Patients exhibiting clinical characteristics such as purity, scaly, pigmented papules in the interscapular area are also suspected of having MEN2A, as these characteristics are linked to CLA.

Patients with characteristic clinical symptoms of MEN2 should be evaluated:

- to reduce overall morbidity and death, searching for additional common malignancies that may be linked with it,
- to perform genetic mutation screening in order to test additional family members and offer them appropriate preventive medical and surgical care, such as a prophylactic thyroidectomy.

Sporadic MTC shares the same clinical appearance as hereditary MTC, with the exception that the latter manifests early in life. This is why the patient has to have a fine-needle aspiration (FNA) biopsy in order to diagnose MTC, and first-degree relatives as well as the patient should be genotyped for any underlying possible RET mutations. When a patient is asymptomatic and has a high blood calcitonin level or undergoes genetic testing (to check for a positive family history), MTC is frequently detected in a preneoplastic condition, such as neoplastic C-cell hyperplasia [23].

In MEN2, the likelihood of PHEO developing before MTC is lower. Following a diagnosis of PHEO, all patients need to have further evaluations for related tumors, including RET genotyping and appropriate biochemical and radiological screening. Since there is no correlation with MEN2B and less than 20% with MEN2A, PHPT alone does not warrant further testing [21].

#### 2.1.3. Diagnosis

When an index patient—the first member of the family to be affected—has a particular kind of mutation—high risk, moderate risk, or low risk—genetically testing for the *RET* protooncogene is used to diagnose and identify the mutation [18].

The kind of mutation determines not only the expressivity and penetrance of the illness but also when to begin the screening process for related cancers and when to perform preventative surgery by avoiding the need to test for every conceivable mutation in every member of the family. Testing for the most often mutated codons in exons 10 and 11 is the first step in the assessment process for an index patient with suspected MEN2A. If this test is negative, we then seek for other frequently mutated codons in descending order. In the absence of a germline mutation, the risk of MTC is minimal. One possibility in this situation is to sequence the whole *RET* coding area in order to find a *RET* mutation. The index patient with the MEN2B phenotype receives the same treatment. Initially, the patient is examined for frequent mutations, such as the A883F mutation in exon 15 if the results are negative, and for the mutation in the *RET* codon M918T in exon 16. MEN2B instances involving these two mutations account for almost 95% of cases. Sequencing the complete *RET* coding region is recommended if no mutations are found.

Additional situations where genetic testing could be useful include:

- First-degree relatives who have a documented germline *RET* mutation.
- Parents whose newborns or young toddlers have MEN clinical symptoms.
- Families with young toddlers or newborns suffering from Hirschsprung disease.
- Individuals with CLA [1].

Since *RET* genotyping only requires a tiny blood sample, it may be carried out at birth or shortly thereafter. Genotyping should be completed as soon as possible to allow for the possibility of a preemptive thyroidectomy in the case of a positive result.

Young children with certain *RET* mutations may experience the onset of clinically noticeable MTC. Screening for known *RET* mutations without clinically evident illness aims to screen for the possibility of prophylactic thyroidectomy before MTC manifests or while the disease is still limited to the thyroid gland. Monitoring begins at age three for children who test positive for high-risk mutations and at age five for those who test positive for moderate-risk mutations. The ages for prophylactic thyroidectomy and for the screening for MTC are listed in Table 2, adapted from [18].

When index patients or asymptomatic individuals with a positive family history have positive *RET* genotyping, biochemical and imagistic screening for additional malignancies is initiated. Moreover, an annual cervical ultrasonography and serum calcitonin dosage are performed for screening.

Additionally, the risk of developing PHEO varies according to genetics. PHEO may be identified early and without symptoms due to screening programs. By the age of 11, yearly PHEO screening should start for kids in the high-risk categories. The yearly screening process for children in the moderate-risk group begins at age 16. Normetanephrines and plasma-fractionated metanephrines, as well as 24 h urine metanephrines assays, are used in screening testing. Adrenal imaging with CT or MRI is the next step if the biochemical results are positive. Adrenal venous sampling might be performed if the first imaging is not able to distinguish between a unilateral and a bilateral adrenal mass. The indicated ages for the screening are listed in Table 3, adapted from [18].

Only linked to MEN2A, hyperparathyroidism is frequently moderate and asymptomatic. One study found that the mean age at diagnosis was 33 years old. For kids at high risk, annual biochemical screening begins at age 11, and for patients at moderate risk, it begins at age 16. Serum calcium adjusted for albumin levels is the preferred screening test. Serum parathyroid hormone (PTH) levels are evaluated if they are raised, and the diagnosis of hypercalcemia is made based on high or abnormally high PTH levels.

#### 2.1.4. Clinical Management

Almost all MEN2A and MEN2B patients experience MTC. The condition, which includes its precursor neoplastic C-cell hyperplasia, is often bilateral and multicentric [38]. Total preventive thyroidectomy with lymph node dissection is recommended in the first year of life for individuals who are predisposed to MEN2B since metastatic disease has been observed in these patients shortly after one year of age. At age five, a complete thyroidectomy with lymph node dissection is recommended for kids with high-risk MEN2A mutations. Metastasis may be discovered early in central and lateral cervical lymph nodes, and MTC is typically associated with the cause of death in MEN2. Lung, liver, and bone are the most common distant metastases, while skin and brain are less common [39,40].

Although the introduction of targeted medicines appears to be increasing progression-free survival in advanced patients, surgery remains the primary treatment and the sole hope for recovery. Significant progress has been made in the treatment of MEN2A since the involvement of RET in the disease was identified, and the majority of children with MEN2A who had early thyroidectomies are now living full and productive lives. Strong connections between genotype and phenotype have made it easier to design intervention guidelines. In addition to the results of genotyping, modern methods for determining the optimal age for surgery include information from calcitonin tests and ultrasound. Children with MTC and MEN2 disorders are best treated in tertiary facilities with comprehensive competence to maximize treatment and support ongoing research.

Moreover, patients with genes that carry a moderate risk may postpone surgery until late adolescence or early adulthood. PHEO must be ruled out before surgery for MTC, and if it is present, it must be removed first.

PHEO develops in around 40% of MEN2A patients and 50% of MEN2B patients, and unlike its sporadic counterparts, it is also likely to be bilateral and multicentric [41]. 

Patients with unilateral adrenal mass or those with bilateral PHEO who have a family history of malignant bilateral tumors are advised to undergo bilateral adrenalectomy. For individuals with unilateral PHEO and contralateral glands that appear normal, unilateral adrenalectomy is advised. This is because patients who underwent unilateral adrenalectomy and subsequently developed a contralateral tumor had a very low risk of metastatic PHEO, and no cases of catecholamine crisis in MEN2 patients have resulted in death.

In addition, adrenal insufficiency following bilateral adrenalectomy is a lifelong condition with higher morbidity and death. Whenever technically possible, adrenal-sparing surgery can be an option. The goal is to remove the PHEO while leaving enough of the cortex (1/3–1/4) intact to permit normal mineralocorticoid and glucocorticoid activity [41,42].

Patients should have preoperative alpha-blockade before unilateral or bilateral adrenalectomy. They should additionally receive glucocorticoid stress treatment while they are waiting to be transferred to the operating room [34].

PHPT is not seen in MEN2B families and only has a 10% to 25% correlation with MEN2A. Considering that asymptomatic people typically have moderate illness that manifests late and is frequently clinically unclear, prophylactic parathyroidectomy is not advised in these cases. Patients who experience increasing levels of hypercalcemia, bone loss, or renal impairment are candidates for surgery. Hence, rather than having the gland removed, the patient is observed every year or two for changes in bone density, creatinine, and serum calcium. The condition is typically multiglandular, and parathyroidectomy is only performed when the illness starts to worsen and develop clinical symptoms. Once more, it is important to screen the patient for concomitant PHEO. If it is found, adrenalectomy is performed prior to parathyroidectomy [35].

Moreover, MEN2A syndrome has been linked to the occurrence of other solid tumors, including malignant paraganglioma [43].

### 2.2. Multiple Endocrine Neoplasia Type 2B (MEN2B)

MEN2B is less common, occurring in 5% of cases. Although the penetrance of MTC and PHEO is similar to that of MEN2A, PHPT will not be present in this subgroup of patients; instead, they will exhibit a diversity of extra-endocrine symptoms [41].

Early diagnosis and prevention are, therefore, essential.

In all cases of MEN2B (100%), it presents as aggressive MTC. Patients with MEN2B identified by genetic screening may benefit from thyroidectomy as early as the neonatal period [18].

Furthermore, among MEN2B patients, PHEO affects around 50% of them, the majority by the age of 30 [41].

Considering that bilateral PHEO may occur sooner than in MEN2A, MEN2B-PHEO should be treated as any other PHEO in MEN2 syndromes. This is further evidence in favor of adrenal-sparing surgery to prevent adrenal insufficiency in younger individuals [26]. 

Mucosal neuromas, which usually affect the lips and tongue, leading to a characteristic face with enlarged lips, and intestinal ganglioneuromas are also included in the MEN2B condition. Patients diagnosed with MEN2B syndrome frequently have digestive issues, such as intestinal ganglioneuromatosis. Megacolon and persistent constipation are two prevalent disturbances of colonic function [44].

Benign lesions, such as those in the mouth, eyes, and submucosa, can also form in addition to malignant tumors. Young patients have little muscle mass, uneven lumps beneath mucosal surfaces, and dry eyes, giving them a highly distinctive marfanoid habitus. Oral nodules that are yellowish and painless are frequently one of the symptoms. In actuality, neuromas are nerve aggregates made up of hyperplastic Schwann cells [19].

Development problems, a reduced upper/lower body ratio, skeletal deformities (kyphoscoliosis or lordosis), joint laxity, and myelinated corneal nerves are additional characteristics of patients with MEN2B. In particular, patients with MEN2B do not show aortic anomalies or ectopia lentis, in contrast to those with Marfan syndrome [41].

## 3. Multiple Endocrine Neoplasia Type 4 (MEN4) Syndrome

Germline mutations in the *MEN1* gene remain undetectable in a notable subset of patients—ranging from 10% to 30%—who exhibit clinical manifestations consistent with MEN1 syndrome. These individuals, classified as MEN1-mutation-negative, may harbor germline MEN1 mutations situated in regions that escape detection by current genetic testing methodologies, such as untranslated, intronic, and regulatory regions. Alternatively, the absence of detectable mutations could be attributed to somatic mosaicism, wherein postzygotic MEN1 mutations occur, or the presence of germline mutations in other genes, such as *CDKN1B*. Additionally, the clinical presentation of multiple tumors in these cases may be coincidental and unrelated to an underlying germline mutation. To unravel the genetic underpinnings of *MEN1*-mutation-negative cases, approaches including candidate gene analysis, whole-genome sequencing (WGS), or whole-exome sequencing (WES) have been employed [8]. 

Initially observed in rats in 2002, a MEN-like syndrome featuring multiple neuroendocrine malignancies, bilateral adrenal and extra-adrenal pheochromocytomas, medullary thyroid neoplasms, parathyroid hyperplasia, and pituitary adenomas was termed MENX. Notably, these animals lacked mutations in MEN1 and RET genes [45].

In 2006, Pellegata et al. identified the causative mutation in humans: an inactivating germline mutation in the *CDKN1B* gene, encoding the tumor suppressor *p27kip1* protein. *CDKN1B* mutations, occurring in 1.5–3.7% of MEN1-like cases without MEN1 or *RET* mutations, result in a truncated, unstable *p27* protein that disrupts cell cycle progression [45].

This syndrome was later reclassified as MEN4 during the 11th International Workshop on Multiple Endocrine Neoplasia [46]. 

Multiple endocrine neoplasia type 4 (MEN4) is defined by the emergence of endocrine tumors, particularly affecting the parathyroid and/or pituitary glands. Its clinical profile often mirrors that of multiple endocrine neoplasia type 1 (MEN1) syndrome. However, recent studies have suggested that MEN4 tends to present in a milder form, with lower incidence and later onset of diagnosis. The average age of the first appearance of an endocrine tumor is 43.5 years (ranging from 5 to 76 years) for MEN4 syndrome, compared to 31.8 years (ranging from 9 to 71 years) for MEN1 syndrome [47].

Hélène Singeisen’s 2023 review on MEN4 syndrome found that it primarily affects women (75% of cases), with a median age of 49.5 years. The study of 48 symptomatic patients revealed 28 *CDKN1B* variants, mostly missense (44%) and frameshift mutations (35%). The parathyroid gland emerged as the most frequently afflicted organ, with a prevalence of 75% among patients, frequently presenting as uniglandular disease in 86% of cases. Involvement of the pituitary gland was observed in 44% of patients, with 76% manifesting hormone-secreting conditions. Additional neoplasms were detected in the adrenal glands (three patients) and thymus (two patients). Initial pathological findings commonly localized to the parathyroid (56% of cases) and pituitary glands (23%). Multi-organ involvement varied from affecting a solitary organ (56% of patients) to encompassing four organs (10% of patients). Overall, MEN4 syndrome is an exceptionally rare condition, with primary hyperparathyroidism being a significant pathology, particularly in the form of uniglandular disease [46].

Currently, there is an absence of universally acknowledged clinical practice guidelines for screening pediatric patients for MEN4 syndrome. However, considering the clinical parallels with MEN1 syndrome, it is posited that screening protocols tailored to MEN1-related conditions may offer a foundational template for guidance. It is pertinent to acknowledge that MEN4 syndrome characteristically presents with a less severe phenotype, reduced penetrance, and delayed tumor onset compared to MEN1 syndrome [48].

## 4. Conclusions

In conclusion, the importance of screening in the management of multiple endocrine neoplasia (MEN) syndromes cannot be overstated. Regular and comprehensive screening protocols, including genetic testing and biochemical assessments, are critical for the early detection and treatment of these complex conditions. As research continues to uncover new MEN syndromes, the prevalence of these disorders is likely to increase. This trend highlights the need for the ongoing development and refinement of screening strategies to detect and manage these newly identified forms. By staying ahead of these emerging challenges, healthcare providers can ensure better patient outcomes and more effective management of MEN syndromes.

## Figures and Tables

**Table 1:** Unveiling the map: screening approaches in MEN1.

Tumor (Estimated Prevalence)	Age to Begin Screening (Years)	Annual Biochemical Tests (Plasma or Serum)	Imaging Test
Parathyroid adenoma (95%)	8	Calcium (or iCa^2+^) PTH	Neck US, Sestamibi—following biochemical confirmation
Gastrinoma (21–70%)	20	Gastrin (±gastric pH)	MRI/EUS/CT abdomen
Insulinoma (20%)	5	Insulin Fasting glucose	MRI/EUS/CT abdomen
Other enteropancreatic tumors (20–70%)	10	CgA, PP, Glucagon, VIP	MRI/EUS/CT abdomen—annually for tumors > 1 cm
Anterior pituitary tumors (15–50%)	5	Prolactin IGF1	2 yearly MRI pituitary
Thymic and bronchial carcinoids (3–10%)	18	None—often lacks secretory activity but can develop malignancy	- Administer a low-dose chest CT scan at age 18 or upon diagnosis - Schedule a low-dose chest CT scan at age 40 - Opt for a biannual chest MRI when a CT scan is not conducted
Adrenal lesions (20%)	10	None unless symptoms develop or identified tumor > 1 cm Renin, aldosterone, U&Es 24 h UFC, overnight dexamethasone suppression test 24 h urinary metanephrines Total testosterone, DHEA-S	2 yearly MRI abdomen

Adapted from [15,16,17]. MEN1: multiple endocrine neoplasia type 1; PTH: parathyroid hormone; iCa^2+^: ionized calcium; neck US: neck ultrasound; EUS: endoscopic ultrasound; CT: computed tomography; MRI: magnetic resonance imaging; CgA: chromogranin A; PP: pancreatic polypeptide; VIP: vasointestinal polypeptide; IGF1: insulin like growth factor I; U: urea; Es: electrolytes; UFC: urinary free cortisol; DHEA-S: dehydroepiandrosteron sulphate.

**Table 2:** The ages for prophylactic thyroidectomy and for the screening for MTC.

Risk	*RET* Mutation	Age at Which It Is Recommended to Start Annual Screening for MTC	Age at Which Prophylactic Thyroidectomy Is Recommended
Highest	918	-	In the first year of life
High	634, 883	3 years	At or before 5 years
Moderate	533, 609, 611, 618, 620, 630, 666, 768, 790, 804, 891, 912	5 years	Childhood or young adulthood

MTC: medullary thyroid cancer.

**Table 3:** The indicated ages for the screening.

Risk	*RET* Mutation	PHEO Age at Which It Is Recommended to Start Annual Screening	PHPT Age at Which It Is Recommended to Start Annual Screening
Highest	918	11 years	-
High	634, 883	11 years	11 years
Moderate	533, 609, 611, 618, 620, 630, 666, 768, 790, 804, 891, 912	16 years	16 years

PHEO: pheochromocytoma; PHPT: primary hyperparathyroidism.
